# Non-leisure time physical activity is an independent predictor of longevity for a Taiwanese elderly population: an eight-year follow-up study

**DOI:** 10.1186/1471-2458-11-428

**Published:** 2011-06-03

**Authors:** Yu-Pei Lin, Ying-Hsiang Huang, Feng-Hwa Lu, Jin-Shang Wu, Chih-Jen Chang, Yi-Ching Yang

**Affiliations:** 1 Department of Family Medicine, Kaohsiung Municipal Min-Shen Hospital, Kaohsiung, Taiwan; 2 Department of Family Medicine, National Cheng Kung University Hospital, Tainan, Taiwan; 3 Department of Medicine, College of Medicine, National Cheng Kung University, Tainan, Taiwan

## Abstract

**Background:**

The aim of this study is to determine the relationship between leisure time physical activity (LTPA) and non-leisure time physical activity (NLTPA) on mortality among the elderly in Taiwan.

**Methods:**

This is a prospective observational cohort study. We analyzed the mortality data from a cohort of 876 non-institutionalized community-dwelling men and women aged 65 years or over, who were recruited by stratified clustering random sampling from Tainan city and participated in the 1996 Elderly Medication Survey. Information about activities and other variables were collected by structured interviews at baseline in the participants' home. The Cox proportional hazards model and crude death rate were applied to estimate mortality risk.

**Results:**

Among the 876 participants, 312 died during the follow-up period (1996-2004). In the unadjusted Cox regression model, subjects aged over 75, having difficulty in carrying out activities of daily living (ADLs), a BMI less than 18.5, a history of diabetes mellitus or stroke, without LTPA or being inactive in NLTPA, were found to have a higher risk of eight-year mortality. With the adjustment for age, gender, education level, habitual smoking and drinking, living status, BMI and medical history, the mortality was found to be higher among the sedentary subjects, either defined by lack of LTPA or NLTPA, with the hazard ratio of 1.27 (95% confidence interval [CI] = 0.97-1.66) and 1.45 (95% CI = 1.07-1.97), respectively. Furthermore, when both LTPA and NLTPA were put into the model simultaneously, NLTPA (HR = 1.40; 95% CI = 1.03-1.91) but not LTPA (HR = 1.21, 95% CI = 0.92-1.59) significantly predicted mortality during eight-year follow-up. In addition, subjects who were actively engaged in NLTPA had a lower mortality risk especially in subjects without performing LTPA.

**Conclusions:**

NLTPA is an independent predictor of longevity among older people in Taiwan. A physically active lifestyle, especially engaged in NLTPA, is associated with lower mortality risk in the elderly population. We thus suggest that encouraging older people to keep on engaging in customary NLTPA is good for their health.

## Background

Regular physical activity is a modifiable factor for reducing the risks of obesity, cardiovascular disease, hypertension, diabetes, certain cancers, and premature mortality, and all of these benefits have been demonstrated in the elderly [1-8]. Physical activity has been defined as "any bodily movement produced by skeletal muscle and resulting in energy expenditure" [9]. LTPA is defined as "physical activity performed during exercise, recreation or any time other than those associated with one's regular occupation, housework, or transportation", while NLTPA are those associated with transportation, occupations, and housework [10]. However, most of the related studies focus only on leisure time physical activity (LTPA), and have less emphasis on non-leisure time physical activity (NLTPA), which comprises the majority of physical activity for the elderly [11-16].

Research shows that NLTPA is the major source of overall physical activity among the elderly. The National Human Activity Pattern Survey (NHAPS), which included representative samples from the United States, revealed that housework related activities contributed 35.2% of total energy expenditure compared to 5.2% of leisure time physical activities in subjects aged 65-74 [11]. A 10-year follow up study with 1,042 people aged 65 years or above conducted at Nottingham, the UK, reported that the time spent on housework is four to eleven times as much as that spent on leisure activities [12]. In addition, the prevalence of individuals achieving the recommended levels of physical activity increased 1.3 to 3-fold after including housework [13,14]. In a large survey of elderly British women, the percentage that reached the recommended levels increased from 21% to 66.7% if housework was included [15]. Focusing only on LTPA may thus underestimate the total amount of physical activity and bias the relationship between physical activity and mortality in women, whose NLTPA constitutes 82% of their daily activity [17,18]. Similarly, in the elderly NLTPA is the major source of overall physical activity.

While the benefits of LTPA with regard to elderly mortality are well-documented in the literature [1-8], the effects of NLTPA have not been studied as extensively [12,18-20]. Glass et al. found that NLTPA was associated with a lower mortality rate in a community-dwelling older population [19]. Morgan et al found that NLTPA predicted all-cause mortality over 10 years in British adult aged 65 years or over [12]. Arriet and Russel also found that higher levels of NLTPA were associated with reduced risk of all-cause mortality over a 20 year in non-institutionized U.S adults aged 60-74 [20]. In recent years, more researchers have focused their attention on NLTPA and proposed that the current general emphasis on purposeful, structured, recreational activities may be too narrow [21].

The purpose of this paper was thus to determine the relationship between two types of physical activity, namely NLTPA and LTPA, and the mortality risk of community-dwelling elderly Taiwanese in an eight-year follow-up study.

## Methods

### Subjects

Tainan city, located in southern Taiwan, comprises seven administrative districts with more than 710,000 residents in 1996. The sampling scheme was a three-stage process that generated a stratified cluster sample of eligible subjects throughout the city [22]. First, areas of the city were grouped into seven strata according to the administrative districts. One area ("li" in Mandarin) was randomly selected from each stratum. Second, all residents aged 65 years or older from each area were selected. In total, there were 1,435 subjects aged 65 years or above were candidates to be included in our study. Third, the selected subjects were informed about the survey by letter, telephone and door-to-door invitation to ask for consent and to arrange a suitable item for the home visits. This survey had received approval from the Institutional Review Board of National Cheng Kung University Hospital, and the investigations were carried out in accordance with the Helsinki Declaration.

### Data collection

First, we collected as much of the data directly from the study subjects as possible. If our study subject had difficulty in communicating or comprehension (such as hearing or cognitive impairment, mild dementia, and so on), we interviewed the primary caregiver, alternatively. At baseline, information was collected through face-to-face home interviews by a well-trained pharmacist using a structured questionnaire that included items related to demographic data (e.g., age, sex, education level), living status (living alone or not), lifestyle habits (such as smoking and drinking habits), levels of leisure time and non-leisure time physical activity, medical history (including cancer, stroke, heart disease, diabetes, liver disease, renal disease, pulmonary disease, hypertension, and osteoarthropathy) and daily living activities (including bathing, dressing, using the toilet, moving from a bed to a chair and back, eating and voluntarily controlling urinary and fecal discharge). Body height and weight measurements were obtained using standardized protocols.

### Leisure time physical activity assessment

Physical activity was assessed at the beginning of the follow-up period in 1996. Subjects were asked about the frequency and duration of performing twelve common LTPA in the previous two weeks, which were as follows: calisthenics or tai chi, gardening, walking for pleasure, bicycling, jogging, hiking, aerobic dancing, folk dancing, tennis, swimming, golf, and miscellaneous exercise. The intensity levels were estimated using the compendium of physical activities developed by Ainsworth et al [23]. For each individual, LTPA energy expenditure in MET-hours per kilogram body weight can be estimated by summing up the energy expenditure of all activities on a weekly basis (MET·hr·wk^-1^). We divided the subjects into two groups: LTPA non-participants (MET-hour/week = 0) and LTPA participants (MET-hour/week > 0).

### Non-leisure time physical activity assessment

An NLTPA questionnaire was used to assess the level of activity with regard to housework and transportation (see Additional file 1). Based on the Physical Activity Scale for the Elderly [24], which has been validated for assessments of physical activity in epidemiological studies of older and diverse populations, we adapted a modified scale suitable for elderly Taiwanese subjects, and it had been verified in terms of both expert and content validity. The test-retest reliability after two weeks was 0.99. In this analysis, subjects who engaged in any activities of NLTPA (either housework or transportation) at least five times per week were classified as part of the "NLTPA active" group, and those who failed to meet this level as the among the "NLTPA inactive" group. The cut-off point to define NLTPA is arbitrary. We refer the ACSM's (American College of Sports Medicine) exercise guidelines for older adults, which recommended the older adults to perform moderate-intensity physical activity at least 5 times/week.

### Mortality

Vital status was verified by matching the subjects' personal identity numbers with the national death registry file obtained from the Department of Health, Taiwan, where all death certificates issued by doctors are sent. Mortality follow-up began on 1 January 1996, and ended on 31 December 2004.

### Data analyses

The SPSS 17.0 software package was used in the statistical analyses. Adjusted and unadjusted Cox analyses were used to estimate mortality risk for the assorted variables. We excluded subjects who had deficits in their ADLs from the adjusted Cox regression model because difficulty in ADLs represents the presence of limitation in physical activity. The crude death rate, calculated in terms of 1,000 people per year, was used to compare the mortality between different levels of physically activity groups.

## Results

A total of 188 of 1,435 registered residents who were selected for the study were no longer living at their previous address. The response rate among the remaining 1,247 subjects was 70.2% (876 of 1247). Reasons for non-response were "impossible to contact during the three visits" 201(54.2%); "living in other cities temporarily" 128(34.5%); "refusal to be interviewed" 25(6.7%); and "difficulty to be interviewed due to poor health" 17(4.6%). In terms of source of information, 834(95.2%) interviews were responded by the study subjects and the other 42(4.8%) were by primary caregivers. We compared sociodemographic data between responders (n = 876) and non-responders (n = 559). Table 1 shows that responding was not associated with gender or education level. However, non-responders had a statistically significant higher average age and higher prevalence of widow/widower status than the responder group.

**Table 1:** Comparison of sociodemographic data between responders and non-responders in the Elderly Medication Cohort Follow-up Study in Tainan City, Taiwan, 1996-2004

	Responders(%)	Nonresponders(%)	
			
Variables	(N = 876)	(N = 559)	P Value ^a^
Age, years	72 ± 6.1	73.8 ± 7.1	<.005^b^
Age group			<.05
65-69	330 (37.7)	186 (33.3)	
70-74	279 (31.8)	152 (27.2)	
75-79	148 (16.9)	108 (19.3)	
≧80	119 (14.6)	113 (20.2)	
Sex			NS^c^
Men	477 (54.5)	294 (52.6)	
Women	399 (45.5)	265 (47.3)	
Education level			NS
Illiterate	367 (41.9)	223 (39.4)	
Elementary	313 (35.7)	195 (34.9)	
Junior high	79 (9.0)	52 (9.3)	
Senior high	67 (7.6)	56 (10.0)	
≧college	50 (6.7)	33 (5.9)	
Marital			
Married	549 (62.7)	288 (51.5)	
Widow/widower	206 (23.5)	166 (29.9)	
Divorced	21 (2.4)	10 (1.8)	
Unmarried	100 (11.4)	95 (17.0)	
Occupation			NS
Yes	182 (32.2)	225 (40.3)	
No	594 (67.8)	334 (59.7)	

^a ^Chi-square test between responders and nonresponders.

^b ^t test.

^c ^NS = no significant at p value > .05.

Table 2 demonstrates the comparison of characteristics of the participants across LTPA and NLTPA groups. We found that subjects aged under 75 with a higher education level, normal weight (BMI between 18.5 and 30 Kg/m^2^) and without history of stroke were more active in LTPA. Those aged under 75, living alone, normal weighted, without ADLs deficit and no history of stroke or arthritis had a higher percentage that engaged in NLTPA.

**Table 2:** Comparison of baseline variables in LTPA and NLTPA groups in the Elderly Medication Cohort Follow-up Study in Tainan City, 1996-2004

	LTPA^1^			NLTPA^2^		
		
	Participants (n = 593)	Non-participants (n = 283)	P^3^	Active (n = 697)	Inactive (n = 179)	P^3^
Age(y/o)			<0.01			<0.01
65-75	68.5	27.1		69.4	40.8	
76-80	17.0	32.2		15.2	24.0	
≧81	14.5	29.7		15.4	35.2	
Sex			0.19			0.94
Male	56.0	51.2		54.5	54.2	
Female	44.0	48.8		45.5	45.8	
Education level			<0.01			0.22
Under elementary school	27.0	18.4		25.1	20.7	
Junior high school and above	73.0	81.6		74.9	79.3	
Smoking status			0.56			0.09
Never/ex-smokers	76.7	74.9		74.9	81.0	
Current smokers	23.3	25.1		25.1	19.0	
Drinking habit			0.06			0.04
< 3 times per week	92.9	96.1		93.1	97.2	
≧3 times per week	7.1	3.9		6.9	2.8	
Living alone			0.13			<0.01
No	90.6	93.6		89.8	98.3	
Yes	9.4	6.4		10.2	1.7	
LTPA						<0.01
Participants	-	-	-	75.5	37.4	
Non-participants	-	-	-	24.5	62.6	
NLTPA			<0.01			
Active	88.7	60.4		-	-	
Inactive	11.3	39.6		-	-	
BMI(Kg/m^2^)			<0.01			<0.01
18.5 ≦ BMI ≦ 30	84.5	63.6		83.2	56.4	
BMI < 18.5	12.8	33.2		13.6	41.9	
BMI < 30	2.7	3.2		3.2	1.7	
ADL			<0.01			<0.01
Without difficulty	99.2	78.1		99.7	63.7	
With difficulty	0.8	21.9		0.3	36.3	
Cancer			0.52			0.13
No	98.5	99.3		98.4	100	
Yes	1.5	0.7		1.6	0	
Hypertension			0.39			0.23
No	68.1	71.0		70.0	65.4	
Yes	31.9	29.0		30.0	34.6	
Diabetes Mellitus			0.31			0.97
No	87.0	89.4		87.8	87.7	
Yes	13.0	10.6		12.2	12.3	
Stroke			<0.01			<0.01
No	96.0	89.4		97.4	79.9	
Yes	4.0	10.6		2.6	20.1	
Heart Disease			0.11			0.82
No	85.0	88.3		85.9	86.6	
Yes	15.0	11.7		14.1	13.4	
Pulmonary disease			0.01			0.56
No	94.6	90.1		93.4	92.2	
Yes	5.4	9.9		6.6	7.8	
Liver disease			0.20			0.13
No	93.6	95.8		93.6	96.6	
Yes	6.4	4.2		6.3	3.4	
Renal disease			0.17			0.46
No	97.5	95.8		96.7	97.8	
Yes	2.5	4.2		3.3	2.2	
Arthritis/osteoporosis			0.01			<0.01
No	63.2	53.4		62.6	50.3	
Yes	36.8	46.6		37.4	49.7	

^1 ^LTPA = leisure time physical activity

^2 ^NLTPA = non-leisure time physical activity

^3^chi-square test for categorical variables

Among the 876 participants, 312 died during the follow-up period (1996-2004). Table3 shows the univariate analysis of all-cause mortality on baseline variables. Subjects who did not participate in LTPA (0 MET-hour/week) had a higher mortality rate than those who were regular LTPA participants (HR = 1.56; 95% CI = 1.24-1.96). Those who were less engaged in NLTPA, that is performed NLTPA less than five times per week, had significantly higher mortality risk than their counterparts who regularly engaged in NLTPA (HR = 2.19; 95% CI = 1.73-2.79). Higher mortality rates were also observed among those subjects who had any difficulty in ADLs (HR = 2.38; 95% CI = 1.71-3.32), BMI less than 18.5 (HR = 1.73; 95% CI = 1.34-2.23), diabetes mellitus (HR = 1.52; 95% CI = 1.12-2.06) or stroke (HR = 1.91; 95% CI = 1.32-2.77) at baseline. Table4 shows the adjusted HR for all-cause mortality after inclusion of either LTPA (model 1) or NLTPA (model 2) separately, and both types of physical activities together (model 3). All three models were adjusted for other 16 covariates such as age, gender, level of education, habitual smoking and drinking, living status, BMI, and medical history including cancer, stroke, heart disease, diabetes, liver disease, renal disease, pulmonary disease, hypertension, and osteoarthropathy. Those subjects who were inactive in LTPA or NLTPA had higher mortality rates with the HR of 1.27 (95% CI = 0.97-1.66) and 1.45 (95% CI = 1.07-1.97), respectively, in models 1 and 2. However, if both NLTPA and LTPA were put into the model simultaneously (model 3), NLTPA but not LTPA could significantly predict mortality. That is, with the control of LTPA and other covariates, subjects who engaged in less NLTPA had a higher mortality rate than their counterparts with the HR of 1.40 (95% CI = 1.03-1.91). To measure the predictive capacity (discriminatory power) of different Cox models, we use the concordance (C-) index (25). There are similar c-index within three models with the value of 0.49 (95%CI = 0.37-0.61), 0.49 (95%CI = 0.37-0.61) and 0.48 (95%CI = 0.37-0.60) in model 1, 2, and 3, respectively.

**Table 3:** Univariate analysis of mortality risk on baseline variables in the Elderly Medication Cohort Follow-up Study in Tainan City, Taiwan, 1996-2004.

**variables**		**Deaths/persons^a^**	**No. of person-year**	**Death rate** **(per 1,000 person-year)**	**HR (95% CI)**
					
Age (y/o)	65-75	86/557	4641	18.5	1
	76-80	96/149	1015	94.6	5.5 (4.1 - 7.4)
	≧81	129/170	1127	114.5	6.7 (5.1 - 8.9)
Sex	Men	179/477	3652	49.0	1
	Women	132/399	3131	42.2	0.9 (0.7 - 1.1)
Educational	Under elementary school	67/212	1691	39.6	1
	Junior high school and above	244/664	5092	47.9	1.2 (0.9 - 1.6)
Smoking	Never/ex-smokers	234/667	5167	45.3	1
	Current smokers	77/209	1616	47.7	1.0 (0.7 - 1.2)
Drinking habit	<3 times/week	293/823	6355	46.1	1
	≧3 times/week	18/53	428	42.1	0.9 (0.6 - 1.5)
Living alone	No	281/802	6365	44.2	1
	Yes	30/74	558	53.8	1.2 (0.8 - 1.8)
ADLs	Without difficulty	271/809	6365	42.6	1
	With difficulty	40/67	418	95.7	2.4 (1.7 - 3.3)
LTPA	Participants	187/593	4730	39.5	1
	Non-participants	124/283	2053	60.4	1.6 (1.2 - 2.0)
NLTPA	Active	213/697	5567	38.3	1
	Inactive	98/179	1216	80.6	2.2 (1.7 - 2.8)
BMI	18.5 ≦ BMI ≦ 30	220/681	5381	40.9	1
	BMI < 18.5	82/170	1192	68.8	1.7 (1.3 - 2.2)
	BMI > 30	9/25	210	42.9	1.0 (0.5 - 2.0)
Cancer	No	305/865	6712	45.4	1
	Yes	6/11	71	84.5	1.9 (0.9 - 4.3)
Hypertension	No	204/605	4696	43.4	1
	Yes	107/271	2087	51.3	1.2 (0.9 - 1.5)
Diabetes mellitus	No	261/769	6012	43.4	1
	Yes	50/107	771	64.9	1.5 (1.1 - 2.1)
Stroke	No	280/822	6401	43.7	1
	Yes	31/54	382	81.2	1.9 (1.3 - 2.8)
Heart disease	No	262/754	5840	44.9	1
	Yes	49/122	943	52.0	1.2 (0.9 - 1.6)
Pulmonary disease	No	289/816	6315	45.8	1
	Yes	22/60	468	47.0	1.0 (0.7 - 1.6)
Liver disease	No	287/826	6409	44.8	1
	Yes	24/50	374	64.2	1.4 (1.0 - 2.2)
Renal disease	No	300/849	6571	45.7	1
	Yes	11/27	212	51.9	1.1 (0.6 - 2.1)
Arthritis/osteoporosis	No	187/526	4069	46.0	1
	Yes	124/350	2714	45.7	1.0 (0.8 - 1.3)

^a ^number of persons at baseline

HR: hazard ratio, ADLs: Activities of daily living, LTPA: leisure time physical activity, NLTPA: non-leisure time physical activity.

**Table 4:** The adjusted hazard ratio for all-cause mortality for sedentary versus active subjects in the Elderly Medication Cohort Follow-up Study in Tainan, Taiwan, 1996-2004.

	Model 1	Model 2	Model 3
			
Physical activities	HR(95%CI)	HR(95%CI)	HR(95%CI)
LTPA			
Non-participants vs. participants	1.27(0.97-1.66)	-	1.21 (0.92 - 1.59)
NLTPA			
Inactive vs. active	-	1.45(1.07 - 1.97)	1.40 (1.03-1.91)
C statistic^a^	0.49	0.49	0.48

67 participants with difficulty in ADLs were excluded in multiple-adjusted Cox model.

Model 1: include LTPA and other 16 covariates (age, gender, level of education, habitual smoking and drinking, living status, BMI, cancer, stroke, heart disease, diabetes, liver disease, renal disease, pulmonary disease, hypertension, and osteoarthropathy).

Model 2: include NLTPA and other 16 covariates.

Model 3: include LTPA, NLTPA and 16 covariates.

We further explored the interaction between LTPA and NLTPA on mortality by comparing hazard ratios of inactive vs active NLTPA groups with different level of LTPA. The results demonstrate that the NLTPA inactive group had a higher mortality risk in both LTPA participant and non-participant groups but the difference is slightly prominent in LTPA non-participant group. The direction is the same and the difference of the effect sizes of NLTPA in the two LTPA strata is not different from a statistically point of view (data not shown).

## Discussion

The main finding of our study is that NLTPA is independently associated with lower mortality risk among elderly Taiwanese, even after adjusting for age, gender, educational level, smoking and drinking habits, living status, medical history, BMI and LTPA. Subjects who engaged in more NLTPA had lower mortality risk especially in the less LTPA group. In our study, NLTPA can predict the survival of older people, and its predictive power was no less than that of LTPA.

Our results are consistent with three cohort studies which suggest that NLTPA is independently associated with lower all-cause mortality among the elderly[12,19,20]. In the United States, Glass et al. conducted a 13-year study on elderly subjects in New Heaven which showed that engagement in productive activities, such as gardening, preparing meals, and shopping, could reduce the mortality risk as much as fitness activities did with adjustment of sociodemographic variables, functional disability, and medical history[19]. Morgan et al. also demonstrated a positive influence of customary activities, predominantly NLTPA (including indoor and outdoor housework, shopping, purposeful walking and so on) on 10-year survival among British subjects aged 65 years or over[12]. Arrieta and Russell estimated that mortality risk reductions were 34% and 38% for moderate and high NLTPA respectively, compared with low NLTPA in person aged 60-74 over a 20-year period[20]. Arrieta and Russell's study was derived from the National Health and Nutrition Examination Survey (NHANES I, 1971-1975) and its Epidemiologic Followup Study NHEFS, a large, nationally representative sample of non-institutionalized U.S. adults aged 35-74 at baseline.

Although the exact mechanisms relating NLTPA to longevity are not well known, several hypotheses have been suggested, and psychosocial pathways are thought to be in operation in addition to direct physical functioning. The psychological effects of NLTPA, such as the maintenance of normal mental states, distraction from negative emotions, and a sense of self-mastery, are proposed to have a positive health impact that goes beyond the benefit of fitness[19,26]. Engagement in social and productive activities may enhance the sense of having a meaningful social role, which then promotes a feeling of self-efficacy and social support. In addition, purposeful social roles have been found to be associated with decreased mortality in later life[27,28]. Secondly, increasing evidence suggests that total energy expenditure through any activity, rather than a specific level of intensity, may confer a survival advantage in older adults[1,17,29]. For example, Manni et al. showed that community-dwelling older adults experienced a 32% lower risk of mortality for every 287 kcal/day in free-living activity energy expenditure[1]. Short bouts of activities, like NLTPA, can be achieved easily by most elderly people. Finally, the beneficial effects of doing housework may be similar to occupational therapy, which can prevent disability and promote quality-of-life in older adults[30].

Because the content of NLTPA is culturally sensitive, research on different ethnic groups is necessary. For example, washing clothes by hand is a common activity for the elderly in our study, but is less common in the West. Previous studies provide tentative evidence for a link between NLTPA and elderly longevity among British and American subjects[12,19]. This study has examined the effects of productive and transportation activities on the mortality risk among Taiwanese elderly, and the results corroborate the earlier findings that increased physical activity, including NLTPA, can decrease the mortality rate in the elderly.

There are several limitations associated with this observational prospective study that warrant consideration. First, there is a potential non-response bias, since the non-responders were older and had a higher prevalence of widow/widower status, and also might be expected to have a higher mortality rate and lower participation in NLTPA and LTPA. Therefore, our results might under-estimate the negative association between NLTPA and mortality in this relatively younger elderly population. Second, some information bias might be inevitable when communication problems occurred in the interviews, perhaps due to hearing or cognitive impairment, which might lead to a non-differentiated misclassification of our data collection. Such problems may have underestimated the real excess risk. Third, we only semi-quantify the amount of NLTPA by frequency of activities, and thus results from this might not be very precise. If additional components of NLTPA, such as duration or amount, are available, the associations between activity and mortality might be more apparent. Further, the information about physical activities was collected for only the two weeks before the interview, and therefore misclassification might occur if the subjects had recently changed their lifestyle or health status. However, such misclassifications may weaken the apparent survival advantage of physical activities, so the real benefits will be more significant than seen in the present analysis. Since the residual confounders cannot be completely eliminated by multiple risk factors adjustments, the results of this study are not sufficient to infer causal relation between survival and NLTPA/LTPA. Finally, three Cox models failed to provide a good discriminatory power with a low C statistic-index. This may be due to the relatively small sample size and other unmeasured mortality risk factors.

The strengths of our study include that we provided a simple and useful culture-sensitive questionnaire to predict eight-year mortality of elderly Taiwanese subjects by level of NLTPA. The six-item questionnaire can be applied in community-based screening programs and identify the highest risk group among sedentary elderly who are engaged in neither NLTPA nor LTPA. Furthermore, all our data were collected by a qualified interviewer, and this minimized the measurement bias.

Our findings have several important implications. In research, the unique relationships between different types of physical activity and mortality underscore the necessity of including multiple domains of activity in epidemiologic studies. In health policy, we can identify the people in the greatest need of intervention among the sedentary elderly. According to the Taiwan National Health Interview Survey, up to 51.4% of elderly men and 42.2% of elderly women never exercise regularly[31]. As a public health priority, we should categorize those individuals who do not engage in NLTPA among the irregular exercise group as a high-risk group of interest for intervention. In practice, healthcare professionals might also recommend a broader range of activities for older people, and encourage them to keep engaging in housework if they already do so. However, in Taiwanese social convention, it is not considered filial if children allow their elderly parents to do housework. We thus propose that the public health messages related to physical activity in this context must emphasize a broader range of activities for older people.

## Conclusions

This study suggests that NLTPA is independently associated with lower mortality risk among the elderly. A physically active lifestyle, engaging in customary NLTPA is good for longevity among the elderly.

## Competing interests

The authors declare that they have no competing interests.

## Authors' contributions

YPL performed study design, statistical analyses, interpreted results and drafted the manuscript. FHL contributed to designing the study and interpreting results. YHH, JSH, and CJC participated in interpreting results and technical support. YCY participated in the study design, statistical analysis, interpretation of data and helped to draft the manuscription. All authors read and approved the final manuscript.

## Pre-publication history

The pre-publication history for this paper can be accessed here:

http://www.biomedcentral.com/1471-2458/11/428/prepub

## Supplementary Material

Additional File **Appendix 1 shows the questionnaire used to assess the level of non-leisure time physical activity**. Click here for file
