# Supplementary material for: Non-leisure time physical activity is an independent predictor of longevity for a Taiwanese elderly population: an eight-year follow-up study
Source: BMC Public Health. 2011 Jun 3;11:428. doi: 10.1186/1471-2458-11-428 (PMC3132163; doi:10.1186/1471-2458-11-428)
Supplement: Additional File — Appendix 1 shows the questionnaire used to assess the level of non-leisure time physical activity. [file 1471-2458-11-428-S1.DOC]

Non-leisure time physical activity is an independent predictor of longevity for a Taiwanese elderly population: an eight-year follow-up study

**Appendix 1: Non-leisure time Physical Activity Questionnaire**

- During the past two weeks, how often did you engage in the following activities?

Option of question No.1 to No.4: 0) <1time/ week 1) 1-2 times/ week 2) 3-5 times/ week 3) >5times/ week

1. Light housework, such as dusting, washing dishes, sewing or carrying out trash.
2. Heavy housework, such as mopping floor, cleaning windows, washing clothes with hands.
3. Cooking or preparing meals
4. Going outdoors for shopping, running errands, visiting friends or relatives

- During the past two weeks:

1. The usual way of outdoor transportation:
   1. never go outside 1) by motor vehicle 2) by bus 3) by bicycle 4) by foot
2. Average flights of stairs climbed:

0) <1 flight/ day 1) 1-5 flights/ day 2) 6-10 flights/ day 3) > 10flights/ day

.
